# Gender differences in binaural speech-evoked auditory brainstem response: are they clinically significant?^☆^

**DOI:** 10.1016/j.bjorl.2018.04.005

**Published:** 2018-05-17

**Authors:** Bahram Jalaei, Mohd Hafiz Afifi Mohd Azmi, Mohd Normani Zakaria

**Affiliations:** a Iran University of Medical Sciences, Faculty of Rehabilitation Sciences, Department of Audiology, Tehran, Iran; b Universiti Sains Malaysia, School of Health Sciences, Audiology and Speech Pathology Programme, Kelantan, Malaysia

**Keywords:** Brainstem, Speech, Binaural, Monaural, Effect size, Tronco encefálico, Fala, Binaural, Monaural, Tamanho do efeito

## Abstract

**Introduction:**

Binaurally evoked auditory evoked potentials have good diagnostic values when testing subjects with central auditory deficits. The literature on speech-evoked auditory brainstem response evoked by binaural stimulation is in fact limited. Gender disparities in speech-evoked auditory brainstem response results have been consistently noted but the magnitude of gender difference has not been reported.

**Objective:**

The present study aimed to compare the magnitude of gender difference in speech-evoked auditory brainstem response results between monaural and binaural stimulations.

**Methods:**

A total of 34 healthy Asian adults aged 19–30 years participated in this comparative study. Eighteen of them were females (mean age = 23.6 ± 2.3 years) and the remaining sixteen were males (mean age = 22.0 ± 2.3 years). For each subject, speech-evoked auditory brainstem response was recorded with the synthesized syllable /da/ presented monaurally and binaurally.

**Results:**

While latencies were not affected (*p* > 0.05), the binaural stimulation produced statistically higher speech-evoked auditory brainstem response amplitudes than the monaural stimulation (*p* < 0.05). As revealed by large effect sizes (*d* > 0.80), substantive gender differences were noted in most of speech-evoked auditory brainstem response peaks for both stimulation modes.

**Conclusion:**

The magnitude of gender difference between the two stimulation modes revealed some distinct patterns. Based on these clinically significant results, gender-specific normative data are highly recommended when using speech-evoked auditory brainstem response for clinical and future applications. The preliminary normative data provided in the present study can serve as the reference for future studies on this test among Asian adults.

## Resumo

**Introdução:**

Potenciais auditivos evocados de modo binaural apresentam bons valores diagnósticos ao testar indivíduos com déficits auditivos centrais. A literatura sobre a resposta do potencial evocado do tronco encefálico com estímulo de fala de modo binaural é, de fato, limitada. As diferenças de gênero nos resultados desse exame têm sido consistentemente observadas, mas a magnitude da diferença de gênero ainda não foi relatada.

**Objetivo:**

Comparar a magnitude da diferença de gênero nos resultados do potencial evocado do tronco encefálico com estímulo de fala entre estímulos monaural e binaural.

**Método:**

Um total de 34 adultos asiáticos saudáveis com idades entre 19 e 30 anos participaram deste estudo comparativo. Dezoito deles eram do sexo feminino (média de idade = 23,6 ± 2,3 anos) e os outros dezesseis do sexo masculino (média de idade = 22,0 ± 2,3 anos). Para cada indivíduo, o potencial evocado do tronco encefálico com estímulo de fala foi registrado com a sílaba sintetizada /da/ apresentada de forma monaural e binaural.

**Resultados:**

Embora as latências não tenham sido afetadas (*p* > 0,05), a estimulação binaural produziu amplitudes de potencial evocado do tronco encefálico com estímulo de fala estatisticamente maiores que a estimulação monaural (*p* < 0,05). Como demonstrado pelos grandes tamanhos de efeito (*d* > 0,80), diferenças substanciais de gênero foram observadas na maioria dos picos de potencial evocado do tronco encefálico com estímulo de fala para ambos os modos de estímulo.

**Conclusão:**

A magnitude da diferença de gênero entre os dois modos de estímulo revelou alguns padrões distintos. Com base nesses resultados clinicamente significantes, os dados normativos específicos para o gênero são altamente recomendados quando se usa o potencial evocado do tronco encefálico com estímulo de fala para aplicações clínicas e futuras. Os dados normativos preliminares fornecidos pelo presente estudo podem servir como referência para futuros estudos sobre esse exame em asiáticos adultos.

## Introduction

Auditory brainstem response (ABR) is a well-known objective test for assessing the neural function within the auditory brainstem region. Commonly evoked by stimuli such as clicks or tone-bursts, it has been used for various clinical applications in the fields of audiology and neuro-otology for many decades. It is found to be useful in estimating hearing sensitivity among difficult-to-test children, newborn hearing screening, neurodiagnostic testing, intraoperative monitoring and so forth.^1^

**Figure 1:**
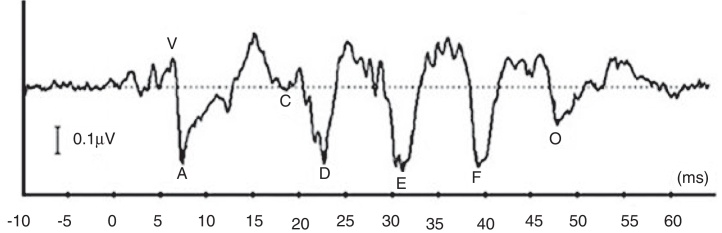
A typical speech-evoked auditory brainstem response (speech-ABR) elicited by a 40 ms speech syllable /da/.

More recently, there is a growing attention to study ABR when evoked by complex stimuli.^2^, ^3^, ^4^, ^5^, ^6^ By utilizing complex stimuli such as speech sounds to record ABR, information on how speech acoustic features are encoded at the subcortical level can be obtained in an objective manner.^2^ This would promote the potential use of ABR in studying subjects with auditory processing deficits involving the brainstem region.^4^ In many studies, a consonant–vowel syllable such as /da/ has been used to record speech-evoked ABR (speech-ABR).^5^, ^6^, ^7^, ^8^, ^9^, ^10^ As depicted in Fig. 1, the speech-ABR typically consists of seven peaks that faithfully describe the acoustic elements of the syllable /da/. The transient element of /da/ is represented by the onset peaks (V and A), while peak C represents the consonant-to-vowel transition segment. The phase-locked sustained component of syllable /da/ is represented by Frequency Following Response (FFR) (peaks D, E, and F) and peak O reflects the offset portion of steady-state vowel. Abnormal speech-ABR results have been reported in subjects with compromised subcortical functions including those with auditory processing disorders, learning disabilities, attention deficit hyperactivity disorder and autism spectrum disorders.^7^, ^8^, ^9^, ^10^

Prior to its clinical applications, the possible influence of demographic factor such as gender on speech-ABR waveforms should be studied. Several studies have reported gender disparities in some speech-ABR results.^1^, ^6^, ^11^ Herein, compared to males, more superior speech-ABR results are found in females. As such, gender-specific normative data can be beneficial for clinical applications.^6^, ^12^ Factors such as anatomical differences and hormonal influence have been discussed for explaining the gender differences in speech-ABR outcomes.^6^, ^11^, ^12^, ^13^ Moreover, most of studies recorded speech-ABR with monaural stimulation and the literature on binaural stimulation of speech-ABR is limited. Studying the binaural sound processing offers several advantages. Firstly, compared to monaural listening, binaural hearing is more realistic as humans use both ears to process and interpret meaningful auditory information. Secondly, the information from both monaural and binaural stimulations of ABR would assist in a better identification of subjects with central auditory disorders.^14^, ^15^, ^16^, ^17^

Ahadi and colleagues were the first to report the gender influence on speech-ABR results with the binaural stimulation.^12^ In their study, significant gender disparities were noted in onset components, composite onset measures and spectral magnitudes of speech-ABR. Nevertheless, the magnitude of gender difference in speech-ABR evoked by monaural and binaural stimulations has not been determined. If strong gender disparities (that are clinically significant) are found in speech-ABR peaks for either mode of stimulation, the relevance of having gender-specific normative data for clinical applications would be even more emphasized. As such, these normative data would be essential for achieving accurate diagnoses when testing individuals with impaired subcortical functions. The present study, therefore, aimed to compare the magnitude of gender difference in speech-ABR results between the two modes of stimulations.

## Methods

### Participants

A total of 34 young Asian adults aged 19–30 years participated in this comparative study. Eighteen of them were females (mean age = 23.6 ± 2.3 years) and the remaining sixteen were males (mean age = 22.0 ± 2.3 years). They were recruited randomly among students and staff members of the respective institution. All of them were healthy and reported no history of ear problems, learning disabilities and neurological problems. Their hearing levels were within the normal limit (pure tone audiometry of 20 dB HL or better between 250 and 8000 Hz) with normal middle ear function (as revealed by type A tympanogram) in both ears. Prior to the data collection, an ethical approval was obtained from the institutional ethics committee (USM/PPP/JEPeM [245.3(5)]), which is in accordance with the 1975 Declaration of Helsinki and its later amendments.

### Equipment and stimuli

Prior to the data collection, all participants provided their informed consent and proper instructions were given. All measurements took place in a sound proof room within the Audiology Clinic, University Hospital. For recording speech-ABR, a two-channel Biologic Navigator Pro AEP system (Natus Medical Inc., Mundelein, USA) was used. The standard 40 ms syllable /da/ (provided by the AEP system) was used to evoke speech-ABR. This stimulus contains an initial noise burst and formant transition between the consonant (/d/) and the vowel (/a/). The fundamental frequency (F0) and the first three formants (F1, F2 and F3) vary linearly (F0 from 103 to 125 Hz, F1 from 220 to 720 Hz, F2 from 1700 to 1240 Hz and F3 from 2580 to 2500 Hz). The last formants, F4 and F5 are constant at 3600 and 4500 Hz, respectively. Four scalp electrodes were placed on the subject's head: non-inverting on the vertex, inverting on each mastoid and ground on the forehead. The impedance of electrodes was kept below 5 kΩ during the testing.

While lying comfortably on the provided bed, the stimulus was presented to each ear (monaurally) and then binaurally through headphones for each subject. The stimulus intensity level was 80 dB SPL and the stimulus rate was 10.9 s. The time window was set at 74.67 ms (including a 10 ms pre-stimulus period). The responses were filtered at 100–1500 Hz and amplified 100,000 times. The recording was stopped when 3584 sweeps were achieved and repeated twice for each trial (to ensure good waveform replicability).

### Statistical analysis

Each peak of speech-ABR waveforms was carefully identified by two competent audiologists. Peak latencies, peak amplitudes and composite onset measures of speech-ABR were then computed for each subject. Both descriptive and inferential statistical analyses were performed as required. Parametric analyses could be performed as all the data were found to be normally distributed (as revealed by Kolmogorov–Smirnov test, *p* > 0.05). To determine the influences of gender and mode of stimulation (monaural vs. binaural) on each speech-ABR result, two-way mixed ANOVA tests (with gender and mode of stimulation as factors) were carried out. To compare between monaural and binaural data, the left and right results were averaged to represent the monaural findings. To provide more detailed information on the effect of each variable on speech-ABR results, paired *t*-test (monaural vs. binaural) and independent *t*-test (male vs. female) analyses were conducted. The statistical significance level was set at *p* < 0.05. To determine the magnitude of gender difference for each stimulation mode, Cohen's effect size (*d*) was computed. The effect sizes were interpreted as small (*d* = 0.20), medium (*d* = 0.50) and large (*d* = 0.80).^18^ All analyses were carried out using SPSS software version 20 (SPSS Inc, Chicago, IL, USA).

## Results

**Figure 2:**
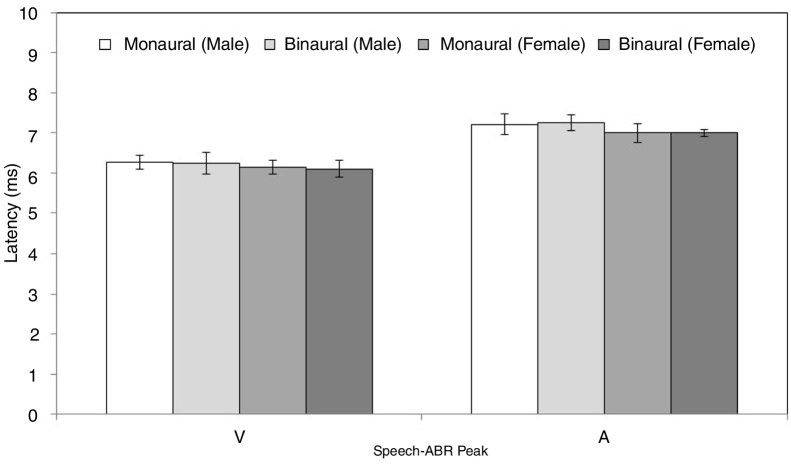
Mean and standard deviation (represented by error bar) of speech-ABR latency (peaks V and A) for each recording condition in male and female participants.

**Figure 3:**
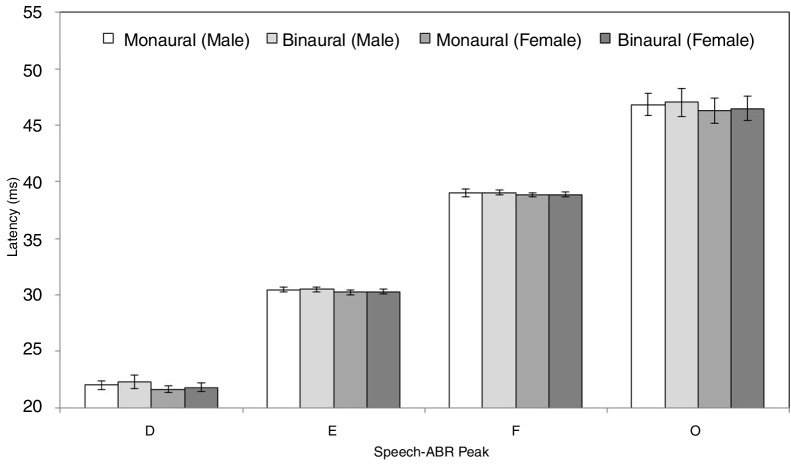
Mean and standard deviation (represented by error bar) of speech-ABR latency (peaks D, E, F and O) for each recording condition in male and female participants.

**Figure 4:**
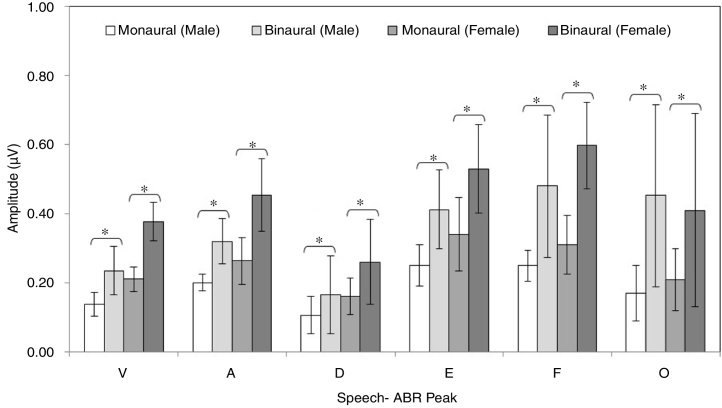
Mean and standard deviation (represented by error bar) of speech-ABR amplitude (peaks V, A, D, E, F and O) for each recording condition in male and female participants (* denotes statistically significant difference at *p* < 0.05).

Figure 2, Figure 3 illustrate the mean latencies of speech-ABR peaks, while the corresponding mean amplitudes are shown in Fig. 4 (for the ease of comparison, the amplitudes for peaks A, D, E, F and O were converted to positive values). As revealed in Figure 2, Figure 3, descriptively, no notable difference in latency was found between monaural and binaural stimulations for each speech-ABR peak in both genders. This observation was then confirmed by the two-way ANOVA results (*p* > 0.05 for all speech-ABR peaks). It is worth noting that since no interaction effects were found in the ANOVA tests for all speech-ABR results (*p* > 0.05), the main effects (mode of stimulation and gender) could be analyzed independently. In terms of speech-ABR amplitudes, for each gender, significantly higher amplitudes were noted in the binaural stimulation compared to the monaural stimulation for all speech-ABR peaks (*p* < 0.05) (Fig. 4). For composite onset measures, the binaural stimulation produced statistically higher V/A amplitudes and steeper V/A slopes than the monaural stimulation for each gender (*p* < 0.05). The V/A duration, on the other hand, was comparable between the two stimulation modes (*p*-values of 0.103 and 0.206 for males and females, respectively).

Table 1, Table 2 reveal the gender comparisons of speech-ABR peaks for the monaural and binaural stimulations, respectively. Generally, it can be seen that the significant *p*-values were supported by large effect sizes. Overall for both modes of stimulations, significant gender disparities (with large effect sizes) were found in most of latencies, amplitudes and composite onset measures of speech-ABR (*p* < 0.05). As revealed, the onset and FFR peaks of speech-ABR were different between genders (Table 1, Table 2). On the other hand, the offset portion (peak O) and V/A duration of speech-ABR results were not statistically different between the two gender groups (*p* > 0.05).

**Table 1:** Mean, standard deviation (SD), 90% range (5th to 95th percentiles), *p*-value and effect size for speech-auditory brainstem response peak amplitudes, latencies and composite onset measures for females and males in the monaural stimulation.

	Female		Male		*p*-value	Effect size (*d*)
	Mean (SD)	90% range	Mean (SD)	90% range		
*Latency (ms)*						
V	6.13 (0.18)	5.89 to 6.38	6.28 (0.17)	5.98 to 6.51	0.021^a^	0.84
A	7.01 (0.24)	6.73 to 7.46	7.21 (0.26)	6.80 to 7.51	0.027^a^	0.80
D	21.58 (0.33)	21.24 to 22.18	21.99 (0.38)	21.50 to 22.69	0.002^a^	1.15
E	30.19 (0.20)	29.93 to 30.53	30.44 (0.21)	30.17 to 30.79	0.001^a^	1.25
F	38.85 (0.18)	38.66 to 39.13	38.97 (0.34)	38.50 to 39.37	0.168	0.48
O	46.31 (1.09)	44.63 to 47.53	46.82 (1.00)	44.88 to 47.79	0.164	0.49

*Amplitude (μV)*						
V	0.21 (0.04)	0.16 to 0.26	0.14 (0.03)	0.09 to 0.17	<0.001^a^	2.08
A	−0.26 (0.07)	−0.35 to −0.18	−0.20 (0.02)	−0.24 to −0.16	0.001^a^	1.25
D	−0.16 (0.05)	−0.23 to −0.10	−0.11 (0.05)	−0.19 to −0.05	0.004^a^	1.06
E	−0.34 (0.11)	−0.46 to −0.16	−0.25 (0.06)	−0.34 to −0.16	0.008^a^	0.98
F	−0.31 (0.09)	−0.43 to −0.19	−0.25 (0.05)	−0.32 to −0.18	0.014^a^	0.91
O	−0.21 (0.09)	−0.42 to −0.12	−0.17 (0.08)	−0.32 to −0.08	0.178	0.48

*Composite onset measures*						
V/A duration (ms)	0.88 (0.11)	0.71 to 1.04	0.93 (0.17)	0.73 to 1.19	0.338	0.33
V/A amplitude (μV)	0.48 (0.09)	0.34 to 0.57	0.34 (0.05)	0.27 to 0.40	<0.001^a^	1.87
V/A slope (μV/ms)	−0.55 (0.14)	−0.68 to −0.31	−0.37 (0.07)	−0.50 to −0.27	<0.001^a^	1.62

a Statistically significant at *p* < 0.05.

**Table 2:** Mean, standard deviation (SD), 90% range (5th to 95th percentiles), *p*-value and effect size for speech-auditory brainstem response peak amplitudes, latencies and composite onset measures for females and males in the binaural stimulation.

	Female		Male		*p*-value	Effect size (*d*)
	Mean (SD)	90% range	Mean SD	90% range		
*Latency (ms)*						
V	6.11 (0.16)	5.90 to 6.35	6.24 (0.17)	5.97 to 6.45	0.025^a^	0.81
A	7.03 (0.21)	6.80 to 7.34	7.26 (0.26)	6.87 to 7.67	0.009^a^	0.94
D	21.80 (0.40)	21.38 to 22.49	22.27 (0.62)	21.76 to 23.47	0.013^a^	0.89
E	30.25 (0.21)	29.98 to 30.59	30.47 (0.22)	30.11 to 30.77	0.007^a^	0.98
F	38.86 (0.18)	38.59 to 39.19	39.02 (0.23)	38.61 to 39.34	0.031^a^	0.77
O	46.53 (1.08)	44.91 to 47.81	47.03 (1.23)	44.55 to 48.20	0.227	0.42

*Amplitude (μV)*						
V	0.38 (0.06)	0.30 to 0.44	0.24 (0.07)	0.13 to 0.34	<0.001^a^	2.25
A	−0.45 (0.10)	−0.58 to −0.28	−0.32 (0.07)	−0.39 to −0.23	<0.001^a^	1.56
D	−0.26 (0.12)	−0.41 to −0.05	−0.16 (0.11)	−0.33 to −0.02	0.033^a^	0.77
E	−0.53 (0.13)	−0.69 to −0.39	−0.41 (0.11)	−0.58 to −0.25	0.011^a^	0.93
F	−0.60 (0.13)	−0.85 to −0.46	−0.48 (0.21)	−0.83 to −0.27	0.044^a^	0.71
O	−0.41 (0.28)	−0.88 to −0.07	−0.45 (0.26)	−0.84 to −0.20	0.688	0.14

*Composite onset measures*						
V/A duration (ms)	0.93 (0.09)	0.77 to 1.03	1.02 (0.19)	0.82 to 1.33	0.093	0.61
V/A amplitude (μV)	0.83 (0.13)	0.63 to 1.02	0.55 (0.10)	0.41 to 0.68	<0.001^a^	2.37
V/A slope (μV/ms)	−0.91 (0.19)	−1.18 to −0.63	−0.56 (0.15)	−0.81 to −0.37	<0.001^a^	2.02

a Statistically significant at *p* < 0.05.

For the monaural stimulation, more significant results (with larger effect sizes) were found in peak amplitudes and composite onset measures than in peak latencies (Table 1). For peak amplitudes, larger effect sizes were observed in the onset portion (*d* values of 2.08 and 1.25 for peaks V and A, respectively) than in the FFR component (*d* = 0.91–1.06). On the contrary, for peak latencies, larger effect sizes were seen in the FFR portion (*d* values of 1.15 and 1.25 for peaks D and E, respectively) than in the onset component of speech-ABR (*d* = 0.84 for peak V and *d* = 0.80 for peak A). No significant difference in the latency of peak F was found between males and females (*t*[32] = 1.411, *p* = 0.168, *d* = 0.48).

Similar to the monaural stimulation, stronger gender differences were also seen in the peak amplitudes than in the peak latencies for the binaural stimulation (Table 2). For peak amplitudes, more pronounced gender disparities were noted in the onset component (*d* = 2.25 for peak V and *d* = 1.56 for peak A) relative to the FFR peaks of speech-ABR (*d* = 0.71–0.93). No such pattern was seen in the peak latencies of speech-ABR. Different from the monaural stimulation, the mean latency of peak F was statistically higher in males than in females for the binaural stimulation (*t*[32] = 2.253, *p* = 0.031, *d* = 0.77). The most prominent gender difference was found in V/A amplitude of speech-ABR (*d* = 2.37) (Table 2). While the gender difference was insignificant in V/A duration (*t*[21] = 1.727, *p* = 0.093), the mean V/A slope was statistically steeper in females than in males (*t*[32] = −5.846, *p* < 0.001, *d* = 2.02).

## Discussion

Recall that the main aim of the present study was to compare the magnitude of gender disparity in the speech-ABR results between monaural and binaural stimulations. The speech-ABR waveforms had been successfully recorded from all subjects. Due to poor detectability, the peak C was not analyzed.

As shown for each gender, the binaural stimulation produced statistically higher peak amplitudes and steeper V/A slopes than the monaural stimulation. These findings are sensible and consistent with the outcomes from previous studies on binaurally evoked speech-ABR and ABR.^19^, ^20^, ^21^, ^22^ Compared to the monaural condition, stimulating both ears would produce greater neural discharge rates resulting in bigger response amplitudes.^23^, ^24^ In the present study, all speech-ABR peak latencies were not affected by the mode of stimulation, which are consistent with the previous studies.^25^, ^26^ In line with this, Ahadi et al. recorded speech-ABR from 48 young adults with left ear, right ear and binaural stimulations.^19^ No significant differences in all speech-ABR peak latencies were found between the left ear and the binaural stimulation. On the other hand, compared to the left ear or binaural stimulation, they found that the right ear stimulation produced significantly shorter peaks A and E. In relation to the present study, such pattern might not be seen as the left and right data were in fact averaged to represent the monaural findings.

For both stimulation modes, significant gender disparities were noted in most of the speech-ABR results. These statistically significant results were then supported by the notable effect sizes. As reported elsewhere, significance testing (with *p*-value) can be controversial and having effect size in the data analysis can be advantageous.^27^, ^28^ Unlike the *p*-value, the magnitude of difference between the tested groups can be determined with the effect size.^27^ Furthermore, it is also less influenced by the sample size. That is, depending on other variables, a substantive difference between the groups can still be noted even if the sample size is small.^28^ The effect size is also among the recommended statistical measures that can be used to determine whether the study outcomes are clinically significant.^29^, ^30^ A large effect size (*d* > 0.80) is indicative of clinically significant results.^30^ In the present study, apart from verifying the *p*-values, the effect size was also used to determine which stimulation mode produced stronger gender effects.

Significant and substantive gender disparities were found in the onset, FFR and composite onset measures of speech-ABR for both stimulation modes. The notable gender differences in speech-ABR onset peak latencies, V/A amplitudes and V/A slopes found in the present study are consistent with the previous reports.^11^, ^12^, ^13^ In line with this, statistically earlier wave V latencies of click-evoked ABR (that correspond to peaks V and A of speech-ABR) were found in women compared to men.^31^, ^32^ Even though controversial, factors such as smaller head size, less brain volume, less skull thickness, shorter fiber tracks, shorter cochlear length and hormonal influence have been suggested as the possible contributors to the robustness of ABR waveforms in females.^1^, ^6^, ^11^, ^12^, ^13^, ^32^, ^33^ It is worth noting that the peak amplitudes and composite onset measures (V/A amplitude and V/A slope) of speech-ABR revealed stronger gender effects than the peak latencies. Since inferential statistical analyses (i.e. significance testing and effect size calculation) on speech-ABR peak amplitudes are not commonly performed in the previous studies, it is therefore difficult to make comparisons. Nevertheless, even though generally amplitudes are more variable than latencies,^1^ the speech-ABR amplitudes can also be useful indicators in gender research, particularly when the testing is conducted in a controlled and optimal condition.

In the present study, significant gender disparities were also found in the FFR component of speech-ABR (peaks D, E and F) for both monaural and binaural stimulation modes. These significant statistical outcomes were supported by large effect sizes, indicating that the gender differences were indeed substantive. These outcomes are not consistent with the findings from studies of Krizman et al. and Ahadi et al., in which the FFR results of speech-ABR were comparable between sexes.^11^, ^12^ The exact reason for this discrepancy is unknown but it may be partly due to subjects’ factor (e.g. sex hormones). In line with the present study outcomes, Liu et al. reported significant gender influences on both onset and FFR portions of speech-ABR recorded monaurally.^13^ Furthermore, in their study on 18 young males and 17 young females, the speech-ABR results were significantly correlated with estradiol and testosterone levels, highlighting the significant influence of sex hormones on speech-ABR waveforms.^13^ In a study by Prabhu et al., Frequency Following Response (FFR) to speech syllable was recorded from 20 young females at four phases of menstrual cycle.^33^ They then found that the FFR responses were influenced by sex hormones (i.e. estrogen and progesterone) as significantly earlier latencies were noted in Phase I (menses) and Phase III (mid-luteal) compared to the other two phases of menstrual cycle. In the present study, more robust FFR waveforms found in females might be related to this hormonal factor. On the other hand, the offset portion (peak O) and V/A duration of speech-ABR were not affected by gender, which are in line with the outcomes of previous studies.^6^, ^11^, ^12^

When the monaural and binaural outcomes were compared, some patterns are observed. That is, while both stimulation modes revealed significant and substantive gender differences, the binaural stimulation produced more pronounced gender disparities in the onset amplitudes (peaks V and A), V/A amplitudes and V/A slopes of speech-ABR. In contrast, as revealed by the larger effect sizes, the gender differences were more prominent in the FFR portion of speech-ABR (peaks D, E and F) for the monaural condition. These findings are rather unexpected and similar outcomes have not been reported in the literature, making the comparison difficult. Further research is therefore required to verify the present study outcomes and shed light on the possible mechanism of this particular aspect of speech-ABR.

The present study, nevertheless, is not without limitations. Firstly, the sample size used was modest and perhaps better study outcomes would be obtained if more subjects could be recruited. Secondly, the spectral analysis of the FFR peaks of speech-ABR could not be performed due to technical problems. Finally, the sex hormones of the participants were not measured and the effect of hormones on binaurally evoked speech-ABR could not be studied.

## Conclusion

By utilizing significance testing and effect size analysis, the magnitude of gender disparity in speech-ABR evoked by monaural and binaural stimulations was studied among young Asian adults. As revealed by large effect sizes, substantive gender differences were noted in most of speech-ABR peaks for both stimulation modes. Based on these clinically significant results, gender-specific normative data are highly recommended when using speech-ABR for clinical applications. The preliminary normative data (including the 90% range) are provided in Table 1, Table 2, which can serve as the reference for future studies on speech-ABR among Asian adults. It is worth noting that similar recording protocols and stimulus parameters should be used in order to utilize these data for specific applications. The usefulness of the normative data can be further studied by recording speech-ABR from subjects with compromised auditory functions. This can be the focus of future speech-ABR research.

## Funding

This work was supported by Research University (RU) Grant (1001/PPSK/812114), 10.13039/501100004595Universiti Sains Malaysia.

## Conflicts of interest

The authors declare no conflicts of interest.
